# When to Stop Antibiotics in the Critically Ill?

**DOI:** 10.3390/antibiotics13030272

**Published:** 2024-03-18

**Authors:** Nathan D. Nielsen, James T. Dean, Elizabeth A. Shald, Andrew Conway Morris, Pedro Povoa, Jeroen Schouten, Nicholas Parchim

**Affiliations:** 1 Division of Pulmonary, Critical Care and Sleep Medicine, University of New Mexico School of Medicine, Albuquerque, NM 87131, USA; jdeaniii@salud.unm.edu; 2 Section of Transfusion Medicine and Therapeutic Pathology, University of New Mexico School of Medicine, Albuquerque, NM 87131, USA; 3 Department of Pharmacy, University of New Mexico Hospital, Albuquerque, NM 87131, USA; eshald@salud.unm.edu; 4 Division of Anaesthesia, Department of Medicine, University of Cambridge, Cambridge CB2 0QQ, UK; ac926@cam.ac.uk; 5 Division of Immunology, Department of Pathology, University of Cambridge, Cambridge CB2 1QP, UK; 6 JVF Intensive Care Unit, Addenbrooke’s Hospital, Cambridge CB2 0QQ, UK; 7 NOVA Medical School, NOVA University of Lisbon, 1169-056 Lisbon, Portugal; pedrorpovoa@gmail.com; 8 Center for Clinical Epidemiology and Research Unit of Clinical Epidemiology, OUH Odense University Hospital, 5000 Odense, Denmark; 9 Department of Intensive Care, Hospital de São Francisco Xavier, CHLO, 1449-005 Lisbon, Portugal; 10 Department of Intensive Care Medicine, Radboud MC, 6525 GA Nijmegen, The Netherlands; jeroen.schouten@radboudumc.nl; 11 Division of Critical Care, Department of Emergency Medicine, University of New Mexico School of Medicine, Albuquerque, NM 87131, USA; nparchim@salud.unm.edu

**Keywords:** antibiotics, biomarkers, antimicrobial resistance, duration of therapy, fixed duration, clinical response

## Abstract

Over the past century, antibiotic usage has skyrocketed in the treatment of critically ill patients. There have been increasing calls to establish guidelines for appropriate treatment and durations of antibiosis. Antibiotic treatment, even when appropriately tailored to the patient and infection, is not without cost. Short term risks—hepatic/renal dysfunction, intermediate effects—concomitant superinfections, and long-term risks—potentiating antimicrobial resistance (AMR), are all possible consequences of antimicrobial administration. These risks are increased by longer periods of treatment and unnecessarily broad treatment courses. Recently, the literature has focused on multiple strategies to determine the appropriate duration of antimicrobial therapy. Further, there is a clinical shift to multi-modal approaches to determine the most suitable timepoint at which to end an antibiotic course. An approach utilising biomarker assays and an inter-disciplinary team of pharmacists, nurses, physicians, and microbiologists appears to be the way forward to develop sound clinical decision-making surrounding antibiotic treatment.

## 1. Introduction

Sepsis remains one of the world’s major killers, with the World Health Organisation estimating 11 million lives lost per year to this condition, with the greatest burden in the global south and a significant burden amongst children [1]. The mainstays of sepsis management, as exemplified by the Surviving Sepsis Campaign guidelines [2], are early diagnosis, early appropriate empiric antibiotic therapy with appropriate source control as appropriate, and organ support. Central to the pathogenesis of sepsis is the infectious agent that triggers a dysregulated host response. For bacterial infections, antibiotics are essential for the elimination of these precipitants. What remains unclear is the optimal duration of antibiotic therapy and how this varies by individual pathogen, infection syndrome, and host response [3,4].

Global antibiotic use has skyrocketed in the 21st century, increasing by 65% between 2000 and 2015, and is on pace to rise by 200% by 2030 if current trends continue [5]. Much of this use has been driven by wider access to antimicrobial agents and an increased recognition of the importance of the early treatment of infection. However, it comes at a considerable cost, perhaps none more threatening than the dramatic global rise in antimicrobial resistance (AMR). AMR is considered to be among the top 10 global health threats, with one study finding 1.27 million deaths worldwide due to AMR in 2019 [6]. While patients with systemic or severe bacterial infections should always be treated with appropriate antibiotics, a potentially modifiable factor for AMR is the prevention of inappropriate prescribing. Unnecessarily long durations of therapy are one major area where the selective pressure of antibiotics could be safely reduced. While the education of clinicians and the public regarding the importance of avoiding unnecessary antibiotic therapy is fairly widespread (although incompletely implemented) [7], an understanding of the optimal duration of antibiotic treatment for a proven or suspected infection is far less prevalent.

The negative consequences arising from the prolonged usage of antibiotics are well described in the literature and vary greatly across the different classes of antibiotics. Aminoglycosides are well known to cause nephrotoxicity and ototoxicity, beta-lactams have been linked to triggering allergic reactions [8], and fluoroquinolones can cause cardiac arrhythmias and QTc prolongation [9], to name just a few common classes. In addition to direct medication side effects, all antibiotics can disrupt the host microbiome, which leads to an increased risk of *Clostridium difficile* infection [10]. The selective pressures acting on potentially drug-resistant organisms occur not just in the wider population but also in the patients themselves, with subsequent infections often resistant to recently administered antimicrobial therapy [11]. It is clear that crafting an appropriate antimicrobial regimen and duration is critical for the optimal treatment of an infection. However, practice in this area ranges widely, and there is little established consensus [7].

Traditionally, clinicians relied on fixed durations of antibiotic administration based on the infection in question or monitored for clinical improvement in vital signs or laboratory values. More recently, biomarkers have been used to guide antibiotic duration and de-escalation. Identifying the optimal approach(es) to guiding antibiotic duration will help minimise antimicrobial-associated harm while ensuring appropriate therapy for bacterial infections. The proposed criteria for guiding the duration of antibiotic therapy are applicable to both documented infections as well as to culture-negative sepsis where the suspicion of infection is high.

## 2. Three Approaches to Determining When to Stop Antibiotics

### 2.1. Fixed Duration

Since the advent of antimicrobial therapy, specific durations of antibiotic therapies have been recommended for certain infections. Longer courses of antibiotics have been the standard of care with the thought that shorter courses may drive the development of antimicrobial resistance. While this notion was endorsed by international health organisations and was the drive behind numerous public health campaigns, these concerns were based only on theory and lacked empirical evidence [12]. In contrast, the “shorter is better” movement was born within the last decade and has taken an evidence-based approach to demonstrate the safety of shorter, fixed-duration antibiotic courses [13]. While the fervour behind this movement is relatively new, the supporting data have been accumulating for years. The evidence supporting the clinical effectiveness of fixed-duration antibiotic courses is robust, and some selected examples are outlined below.

#### 2.1.1. Bacteremia

*Staphylococcus aureus* bacteremia (SAB) is associated with high rates of morbidity and mortality due to the bacteria’s proclivity to spread and stick in deep-seated locations. The recommendation to classify patients into categories based on clinical criteria and subsequently modify therapy duration seemingly originates only from expert opinion [14]. Thereafter, individual investigations have referred to “complicated” and “uncomplicated” SAB, but these definitions have been inconsistent across studies. While this increases the difficulty of interpretation, current professional society guidelines essentially suggest that patients without risk for metastatic sites of infection are candidates for 14 days of therapy after the first negative blood culture, whereas patients with, or at-risk for developing, metastatic disease are treated for four to six weeks after the first negative blood culture. More recent investigations have shifted their focus to subtypes of SAB that may be candidates for abbreviated courses of antimicrobials [15,16].

Gram-negative bacteraemia (GNB) is also associated with high rates of morbidity and mortality. The classical 14-day fixed antimicrobial duration for GNB has been brought into question by the “shorter is better” movement. High-quality investigations of 7- versus 14-day courses of antibiotics required patients to be afebrile and hemodynamically stable for 24 to 48 h to be eligible for randomization to short-course antibiotics. Clinical outcomes were non-inferior in patients who received 7 days of antibiotics as compared to longer courses [17,18,19,20]. Interestingly, the 7-day comparator arm remains within the range of current standard treatment courses, and there may be scope to further reduce the duration of these subsets of infections.

#### 2.1.2. Pneumonia

In several randomized controlled trials of patients with non-severe community-acquired pneumonia (CAP), patients had to achieve clinical stability, determined by features such as apyrexia and hemodynamic stability, prior to randomization. The outcomes of interest—clinical success or cure—were focused on continued stability after antibiotic discontinuation. In these trials, shorter fixed durations of antibiotics (3 to 5 days) were found to be non-inferior to longer courses (8 to 10 days) [21,22,23]. In the case of atypical pneumonia, just one dose of azithromycin was found to be non-inferior to a 3-day course [24].

Similarly, the fixed duration of antibiotics historically used for ventilator-associated pneumonia has been challenged. Unlike the CAP trials that required clinical stability for short-course eligibility, these randomised clinical trials had no such restrictions. Clinical outcomes were found to be similar when patients were treated with a shorter fixed duration of antibiotics compared to a longer course [11,25]. These data were also instrumental in demonstrating that longer courses of antibiotics do not prevent the development of antimicrobial resistance but, conversely, may contribute to increased rates of resistance [11]. However, controversy remains surrounding *Pseudomonas aeruginosa* VAPs, specifically as this microbe has been underrepresented in current studies [26]. Due to the limited data, definitive conclusions about shorter versus longer treatment courses for *Pseudomonas aeruginosa* VAP cannot be drawn.

#### 2.1.3. Intra-Abdominal Infection

Several studies have demonstrated the safety of short courses of antibiotics in patients with severe or complicated intra-abdominal infections. In the STOP-IT trial, for patients with severe or complicated intra-abdominal infections who received adequate source control, antibiotics were continued for a fixed duration (4 days) or for 2 days after resolution of signs of infection (total of 8 to 10 days). Patients treated with shorter fixed-durations of antibiotics had similar clinical cure rates to those treated with more prolonged durations [27,28]. The DURAPOP trial of critically ill patients with intra-abdominal infections similarly showed that shorter courses of antibiotics (8 versus 15 days) were associated with more antibiotic-free days while maintaining similar rates of mortality and length of stay [29].

Like all strategies to determine when to stop antimicrobials, there are pros and cons to utilising the fixed-duration method. An advantage of utilising this strategy is the availability of strong data to support fixed antimicrobial durations in many of the most commonly encountered infections. Additionally, most of the fixed-duration literature has shown similar outcomes for short and long courses of antibiotics. Advocating for shorter courses of antibiotics, when appropriate, may attenuate the development of antimicrobial resistance and adverse drug events [30]. However, short-duration antimicrobial therapy is predicated on effective source control, and if this is not achieved or achievable, such approaches may not be safe. Another consideration is that many, though not all, trials required clinical improvement or stability as an eligibility factor for randomisation to shorter courses of antibiotics. The notion that antibiotics should be continued beyond certain signs of clinical resolution dates back to the 1940s, and its modern clinical relevance is still up for debate (n.b.: this will be discussed further in the next section) [31]. Finally, we must acknowledge the limits of the evidence—the trials and studies completed to date do not cover every bacterial species, infection source, or severity. Indeed, it is important to note that many studies specifically exclude critically ill and immunocompromised patients, and considerable care must be taken in extrapolating existing data to these populations. Although clinicians have historically relied heavily on fixed durations to determine when to stop antimicrobials, the nuances and limitations of this strategy must be respected [32].

### 2.2. Clinical Criteria

For patients and clinicians, the most common indicators of infection are their symptomatic and observable consequences. These typically manifest as pain, altered body temperature, organ dysfunction, and focal signs such as cough or localised swelling. It is these signs that alert us to the presence of infection, and their resolution heralds the end of the illness. Infection is characterised by a host immune response to an invading or overgrowing pathogen [33], and these signs and symptoms arise as a consequence of immune activation or direct pathogen or toxin-mediated damage. Although clinically ‘silent’ or paucisymptomatic infections do occur, these are of limited relevance to critically ill patients unless they lead to secondary infections, as may be seen with human immunodeficiency syndrome and AIDS-defining infections. As these are of limited relevance to antimicrobial therapy duration, they will not be discussed further here. Using clinical criteria to determine the duration of antimicrobial therapy in the critically ill has face validity as an approach to enable personalised treatment. However, clinical signs have to be considered in the context of intercurrent drug therapy, where agents such as glucocorticoids, acetaminophen (paracetamol), or vasopressors may modulate inflammatory signs and symptoms.

Despite this apparently simple and attractive approach, this remains a poorly explored topic, even though intensivists commonly report using it to, at least in part, inform their duration of antimicrobial use [34]. In a systematic review and meta-analysis of antibiotic discontinuation trials, only two clinical algorithm-based trials were identified, and neither demonstrated benefits over fixed or procalcitonin-driven algorithms [35]. Outside of intensive care, discontinuation of antibiotics on clinical grounds appears to be safe and effective. However, for critically ill patients, concerns regarding the severity of infection and the presence of non-infectious mimics of infection may impact the ability of clinical criteria to effectively limit antimicrobial duration.

Antimicrobials commonly eliminate their target pathogens shortly after their initiation, especially where the site of infection is readily accessible to the selected agent. In those with pneumonia, 94% of pathogens are eliminated within 3 days of antimicrobial therapy initiation [36]. Therefore, it is highly likely that antimicrobial durations beyond this are frequently unnecessary. However, the clinical features of pneumonia typically linger longer past the time of bacterial eradication, with improvements in clinical parameters such as temperature and oxygenation usually seen within six days [37]. In an attempt to apply objective criteria to clinical improvement, Singh and colleagues used a modified Clinical Pulmonary Infection Score (CPIS) to guide shortened courses of antibiotics in patients showing clinical improvement [38]. This approach led to a substantial reduction in antibiotic use (3 days vs. 9.8 days) in the intervention arm, with no change in mortality or ICU length of stay, although it was restricted to patients with a CPIS ≤6 (i.e., low probability of VAP) at enrolment and may not be generalizable to those with more definitive features of pneumonia.

In intra-abdominal infections, resolution of fever and normalisation of white cell count at the time of cessation of antibiotics were associated with very low rates of recurrent infection [39], findings that have been replicated in a more recent retrospective cohort observational study [40]. However, of note, in the larger 2006 study by Hedrick and colleagues [40], clinicians using a fixed duration of antibiotics rather than judging the need for them by clinical response tended to use shorter courses and use fewer antibiotics, with no apparent detriment to the patients. Furthermore, in the STOP-IT trial, the use of short (4-day) fixed-duration antimicrobials was non-inferior to a course guided by clinical features, where durations averaged 8 days [27].

As previously noted, one of the issues with clinical features of infection is that they linger after the infecting pathogen has been eliminated. Indeed, this feature of severe infections has been recently and widely illustrated by the COVID-19 pandemic, where respiratory failure and acute respiratory distress syndrome (ARDS) often developed some time after active viral replication ceased [41]. In patients with pneumonia, the lag in resolution is most marked in patients who develop ARDS [42]. Understanding why bacterial eradication and clinical resolution are disconnected is likely critical to understanding and treating organ failure in sepsis. For now, however, it remains a barrier to using clinical features to guide the duration of antimicrobial therapy.

The relative paucity of evidence and lack of randomised trials in this area are reflected in clinical guidelines. In the 2016 update of the American Thoracic Association/Infectious Diseases Society of America VAP and HAP guidelines [43], the authors recommend, albeit with low certainty, against using clinical criteria alone for stopping antibiotics and advise the addition of procalcitonin (PCT). The Joint European/Latin American guidelines for severe CAP also advised the use of PCT to reduce the duration of antibiotic therapy, but noted that clinical features were important and may allow for the earlier (between 5 and 7 days) discontinuation of antibiotics. This may render PCT useless for guiding the duration, though this statement is based upon low-quality evidence [44]. Conversely, the Surgical Infection Society, in their 2017 guidelines, explicitly endorsed using clinical parameters in deciding when to cease antimicrobial therapy in intrabdominal infection [45], despite the evidence from Hendrik and colleagues [40] and the STOP-IT trial [27]. Interestingly, guideline-directed fixed-duration antibiotics may prolong courses beyond what clinicians feel is indicated [46], and thus it is vital that such guidelines are firmly rooted in evidence and acknowledge where this is absent or weak.

Overall, while clinical features are critical to the recognition of infection, inflammation often extends beyond the eradication of the microbes, especially when inflammatory syndromes such as ARDS develop. Furthermore, the assessment of such features can be susceptible to inter-observer variability. Therefore, the use of these features as the sole or predominant metric for limiting antimicrobials is suboptimal and likely to lead to excessively prolonged courses.

### 2.3. Biomarker-Guided

Until the 1990s, the recommended duration of antibiotic therapy for the most common sources of severe infection, such as the lung and abdomen, was 2 to 3 weeks, even though there was little evidence to support these durations [47]. The beginning of the 21st century was marked by two landmark trials [11,38], clearly demonstrating that short courses of antibiotic therapy were not only safe but were equally effective as longer courses. Subsequently, these findings were replicated in different clinical settings (general wards, ICUs, outpatient settings), in different infections, and in different patient populations. As a result, the current recommendations for antibiotic therapy for most ICU infections now range between 7 and 8 days. However, in adopting this practice, we assume that different infections, pathogens, and hosts are all the same and behave similarly. It may well be that 7 to 8 days is too long for some clinical situations or too short for others [48].

As a result of these uncertainties, clinicians, at least in part, have frequently been reluctant to shorten the duration of therapy for severe infections. Could biomarkers help clinicians in this decision-making process?

In recent years, the number of new biomarkers and the frequency of their use in the ICU setting have increased markedly. However, only a few have been assessed as part of biomarker-guided antibiotic strategies.

As with everything in medicine that can be used in clinical decision-making, biomarkers have pros and cons, passionate defenders and obsessive detractors, yet the simple fact remains that the perfect biomarker does not exist [49].

**Figure 1:**
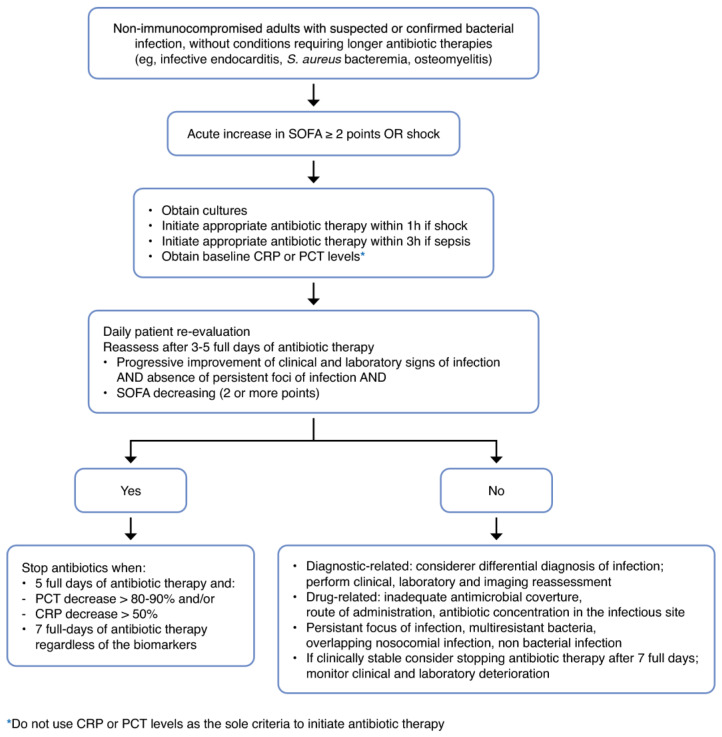
User’s guide for biomarker-guided antibiotic therapy. Starting antibiotics in critically ill patients with suspicion of sepsis should be performed irrespective of any biomarker level, but this should be reassessed daily. The clinical course, the organ dysfunction course (with SOFA score), the kinetics of biomarkers, and the duration of antibiotic therapy should be used to ascertain the optimal duration of therapy. PCT, procalcitonin; CRP, C-reactive protein; SOFA, sequential organ failure assessment, NOTE: CRP and PCT thresholds should be used only as indicative and orientation. These recommendations do not apply to immune-compromised patients nor to patients with infections requiring long-term antibiotic therapy, like endocarditis or osteomyelitis. Reprinted with permission from Ref. [50]. Copyright© 2023, ICM and Springer, Inc.

The most studied biomarkers in infection and sepsis are C-reactive protein (CRP) and procalcitonin (PCT). Observational studies have repeatedly shown that the kinetics of these biomarkers can be used as surrogate markers of response to therapy [50]. These findings have led to the design of algorithms for biomarker-guided antibiotic stewardship (see Figure 1).

PCT-guided antibiotic stewardship algorithms are by far the most studied biomarker-based strategies. Several systematic reviews/meta-analyses (SRMA) have pointed to a decrease in the duration of antibiotic therapy with PCT guidance. The most recent SRMA included 26 RCTs performed in ICUs (N = 9048 patients) and showed, once more, a significant reduction in the duration of antibiotic therapy (on average, 1.79 fewer days) without a negative impact on outcomes [51]. However, this modest impact on the duration of antibiotic therapy (<2 days) was probably attained because in some RCTs the duration of therapy in the control groups was routinely longer than the recommendations based on current evidence [52,53]. In addition, immunocompromised patients and some pathogens (i.e., *Legionella* or *Pseudomonas* spp.) were excluded from some RCTs [48].

There is one SMRA that deserves to be specifically mentioned since it assessed not only the impact of PCT-guided algorithms in the subset of patients with sepsis but also assessed trials according to the rate of algorithm adherence (considered high if >80%) and those using only PCT in the intervention arm (or PCT plus CRP). A decreased duration of antibiotic therapy with PCT-guided algorithms was primarily observed in RCTs with high protocol overruling (low adherence) and in algorithms combining PCT with CRP [54].

Although only one RCT reported mortality as the primary outcome [55], PCT-guided antibiotic stewardship algorithms are overall associated with lower 28-day mortality rates. However, this finding is not universal, and some trials show no mortality impact [51]. In the previously mentioned SRMA [54], the survival benefit was not present in RCTs that only enrolled patients with sepsis, were without industry financial support, had high PCT-guided algorithm adherence, and used PCT-guided algorithms without the addition of CRP. In the most recent SRMA [51], the survival benefit was present only in RCTs using the Sepsis-3 criteria to enrol patients, enrolled “medical” patients, and used so-called “liberal” PCT protocols (i.e., stopping antibiotics if PCT levels were reduced >80% of the peak value or to <0.5 ng/mL). Although antibiotic-related adverse events are widely understood [30], the SRMAs were not able to establish a relationship between shorter antibiotic therapy courses and mortality.

Concerning other clinical outcomes, such as ICU and hospital length of stay or secondary infections, PCT-guided antibiotic stewardship algorithms showed no impact when compared to the standard of care. Moreover, the most recent SRMA indicated a significantly higher risk of recurrent infection in the PCT-guided group [51].

While all the RCTs cited above employed PCT-guided antibiotic stewardship algorithms based upon decreasing PCT levels, the PASS trial assessed the opposite—what to do with non-decreasing daily PCT measurements, the so-called “alert PCT” (a threshold of 1 ng/mL or levels not decreasing by >10%/day) [56]. Patients meeting these criteria were considered “at risk” and consequently underwent protocolized investigation and empiric antibiotic therapy. This approach was associated with higher rates of broad-spectrum antibiotic consumption, more days on antibiotics, prolonged lengths of mechanical ventilation and prolonged ICU lengths of stay, but with no beneficial impact on mortality.

In 2017, the FDA approved PCT to help guide the management of antibiotic therapy for lower respiratory tract infections and sepsis. However, real-world data from several US hospitals, including thousands of patients, have not demonstrated a decrease in antibiotic consumption nor in mortality [57,58,59].

CRP-guided antibiotic stewardship algorithms have been infrequently assessed, and even then only in the adult ICU population. There is only one RCT assessing the impact of a CRP-guided algorithm on antibiotic therapy duration. The CRP group had lower total antibiotic exposure, with a higher rate of antibiotic suspension by day five without a negative impact on other outcomes, including mortality. However, this reduced antibiotic exposure was only related to the index infection episode. There was no reduction in total antibiotic exposure or antibiotic-free days [60].

So far, only one RCT with ICU patients compared PCT-guided with CRP-guided algorithms in a protocol focused on reducing the duration of antibiotic therapy, where the limit was seven days of therapy (fixed duration). In this RCT, CRP was shown to be equally effective as PCT in reducing antibiotic use without any difference in morbidity or mortality [61].

It is worth noting that these last two RCTs were innovative and different from the previous PCT-guided studies in that they used a “double-trigger” strategy [60,61]. Antibiotic discontinuation was recommended according to clinical criteria (as assessed by the SOFA score) and biomarker thresholds (PCT or CRP), or the completion of 7 full days of treatment (fixed duration), whichever came first.

There is one recently published SRMA evaluating CRP-guided antibiotic stewardship algorithms. It included 3 RCTs, of which 2 were performed in the ICU (N = 727 patients). It showed a significant reduction in the duration of antibiotic therapy (on average, 1.82 fewer days) without a negative impact on outcomes, such as mortality or recurrence of infection [62].

It is also important to draw attention to an SRMA evaluating three antibiotic strategies (clinical algorithms, PCT-guided, and fixed duration) that demonstrated that, as compared to fixed duration strategies, PCT-guided algorithms had no added value in further decreasing antibiotic durations [35]. This is reflected in the statement from the recent European/Latin American sCAP guidelines stating that “PCT might not be useful when clinical stability is achieved, and duration of antibiotic therapy is between 5 and 7 days” [44].

Taking into account all the available data and clinical research, the likely optimal approach is to use a multimodal strategy—combining clinical courses (based upon SOFA scores), biomarker-guided algorithms (PCT or CRP), and fixed durations of therapy (around 7–8 days), to attain a more personalised prescription of antibiotics, thereby guiding clinicians to better adhere to antibiotic stewardship programmes, leading to less antibiotic resistance, toxicity, and costs [3,49]. These multimodal strategies could be incorporated into clinical decision support (CDS) systems to help clinicians in the decision-making process at the bedside. Indeed, this strategy is currently being tested in an RCT, with CRP as the primary biomarker (NCT05841875).

## 3. The Final Piece: Implementation

Like in any field of innovation in healthcare, there is a gap between the evidence and the practice of stopping antibiotics. While the evidence—as described above—is increasingly abundant that shorter antimicrobial treatment is safe, including in ICU settings, the literature shows that antibiotics are still often given longer than recommended by guidelines [63,64,65,66].

A better understanding of the factors influencing antibiotic therapy duration among health professionals is needed to develop strategies to effectively address these drivers of duration. A recent systematic review showed that there is only limited literature available that describes factors influencing antibiotic therapy duration. Unfortunately, it showed a complete lack of such studies in the ICU setting [67]. Mostly, the available studies describe differences in therapy duration between certain groups of professionals (e.g., between surgeons and internists) or a for a type of patient or pathogen where duration cannot be determined by specific guideline recommendations. However, these studies do not explain why or how these differences occur [67]. This emphasises the need for ICU-based studies that provide insight into the drivers of professional behaviour regarding the duration of antibiotic therapy.

Stopping antimicrobial therapy or, at least, adhering to guideline recommended durations of therapy is one of the key objectives of an ICU Antimicrobial Stewardship Programme (ASP). Trying to influence intensivists to refrain from starting therapy in the first place has proven to be a challenging experience—after all, who could blame an intensivist who starts antibiotics in an unstable patient with an unclear diagnosis in the deep of night? On the contrary, decision-making about stopping antimicrobial therapy most often takes place in the bright daylight. Studies have discussed the (individual) roles of microbiologists, infectious disease physicians (IDPs), and clinical pharmacists in ICU Antimicrobial Stewardship [4,68] and decision-making regarding the duration of therapy is mostly accomplished during multidisciplinary meetings (MDMs) [69]. A recent qualitative direct observation study showed that in most cases, the ultimate decision on antibiotic therapy duration was the result of multidisciplinary shared decision-making in the MDM. Determining the duration of antibiotic therapy is a senior-level decision in which the intensivist and the clinical microbiologists/IDP were most involved, while ICU residents and referring physicians played a limited role in the decision-making process. When determining the duration of antibiotic therapy in ICU patients, intensivists mostly seemed to focus on the clinical status of the patients, while microbiologists mostly used arguments based on culture results [69].

To improve guideline-adherent antibiotic durations, the first step is to measure compliance. Often, clinicians underrate the duration of therapy they prescribe, but by performing an audit using pharmacy data (e.g., studying days of therapy (DOT) or length of therapy (LOT)), insight into actual durations can be acquired. Then, dependent on a barrier analysis as described in qualitative studies like the one referenced above, a strategy to shorten treatment according to the guidelines may be implemented. A clear local protocol, easily accessible to all prescribers, will help, as will automated stop dates (or at least the obligation to enter a stop date in the electronic prescribing module), but most importantly, a daily evaluation of antibiotic therapy during an MDM will contribute. Daily evaluation, of course, also involves changing therapy (de-escalation) or, where applicable, dose adaptation. The presence of referring physicians (e.g., surgeons) at these meetings to discuss the patient’s clinical condition (e.g., “Has source control been established?”) is pivotal. The use of PCT or other biomarkers (see above) may be integrated into the decision-making process as a useful implementation tool.

## 4. Discussion

As noted above, the data surrounding the adverse impact of widespread antibiotic use are extensive, yet it has only been in recent years that there has been increased interest in revisiting the dogma regarding the duration of antibiotic dosing. To face the growing threat of AMR, we must ensure the optimisation of our antibiotic practices, both with the indication for and the duration of their use. Prior data do indicate a link between increased duration of antibiosis and subsequent risk of AMR. Arulkumaran, et al. demonstrated that a shorter duration of antimicrobial use was associated with reduced AMR rates. Singh, et al. also demonstrated that reduced-duration antimicrobials were associated with fewer secondary infections and fewer AMR infections. Finally, Curran, et al. linked each day of antibiotic therapy with a 4% increased risk of superinfections or AMR [30,35,38]. In this article, we have set out to appraise three common approaches to determining the duration of antibiotic therapy.

The fixed-duration antibiotic strategy presents several advantages, most notably the strong evidence-based support for its effectiveness in various common infections, resulting in similar clinical outcomes for both shorter and longer courses. However, while the evidence base is substantial and growing, it is important to note its limitations. Entry into trials is frequently only permitted once source control is completed and/or clinical stability is achieved. Furthermore, even ‘short’ courses of 7 to 8 days may be too long in some circumstances. Utilising clinical criteria to determine the duration of antimicrobial therapy is also somewhat rooted in the practices of the past, when, with the advent of antibiotics, durations were often terminated at the point of defervescence or other signs of clinical improvement. This approach allows tailoring treatment to the individual patient but risks overlong treatment when inflammation persists after eradication of the pathogen and relies on subjective and variable clinician approaches. Biomarker-guided approaches are the newest approach to the determination of optimal duration. Most of the evidence to date involves the use of procalcitonin and CRP, though other markers are under development and evaluation. The benefits of this approach include objective measurements and less reliance on subjective clinical signs and symptoms, and there is a growing evidence base for their allowing safe shortening of therapy. However, they impose a cost burden not seen with fixed-duration or clinical response, and where the former approaches already achieve short durations of treatment, biomarkers may not achieve much beyond increasing the confidence in these approaches.

While these approaches have been tested in well-conducted, rigorously controlled clinical trials, the implementation of the existing evidence remains patchy. Clinicians often remain anxious about discontinuing antibiotics even in the face of guideline recommendations and evidence, and as we have noted, all of the approaches reviewed contain drawbacks and uncertainties. Perhaps a wider appreciation of the risk of harm to patients from excessively prolonged therapy will bring some balance to this judgement, and as we face an increasingly fraught ‘post-antibiotic’ era, help hold back the tide of AMR organisms and near untreatable infections.
